# New ferrocenyl-containing organic hole-transporting materials for perovskite solar cells in regular (n-i-p) and inverted (p-i-n) architectures†

**DOI:** 10.1039/c8ra08946a

**Published:** 2019-01-02

**Authors:** Jingwen Jia, Liangsheng Duan, Yu Chen, Xueping Zong, Zhe Sun, Quanping Wu, Song Xue

**Affiliations:** Tianjin Key Laboratory of Organic Solar Cells and Photochemical Conversion, School of Chemistry & Chemical Engineering, Tianjin University of Technology Tianjin 300384 PR China cytjut@163.com xuesong@ustc.edu.cn

## Abstract

Three triphenylamine derivatives containing ferrocenyl groups (JW6, JW7 and JW8) were synthesized by facile syntheses. Their HOMO levels match the valence band energy of CH_3_NH_3_PbI_3_. The introduction of ferrocenyl was aimed to obtain hole transporting materials with high mobility for perovskite solar cells. JW7 shows higher hole mobility (4.2 × 10^−4^ cm^2^ V^−1^ s^−1^) than JW6 (1.3 × 10^−4^ cm^2^ V^−1^ s^−1^) and JW8 (1.5 × 10^−4^ cm^2^ V^−1^ s^−1^). Their film-forming properties are affected by their molecule structures. The methoxyl and *N*,*N*-dimethyl terminal substituents of JW7 and JW8 are beneficial for having better solubility than JW6. The regular mesoporous TiO_2_-based perovskite solar cells (n-i-p) and the inverted planar heterojunction perovskite solar cells (p-i-n) fabricated using JW7 show the highest power conversion efficiency of 9.36% and 11.43% under 100 mW cm^−2^ AM1.5G solar illumination. For p-i-n cells, the standard HTM PEDOT-based cell reaches an efficiency of 12.86% under the same conditions.

## Abstract 2

New ferrocene-containing organic HTMs for fabricating perovskite solar cells.

## Graphical abstract

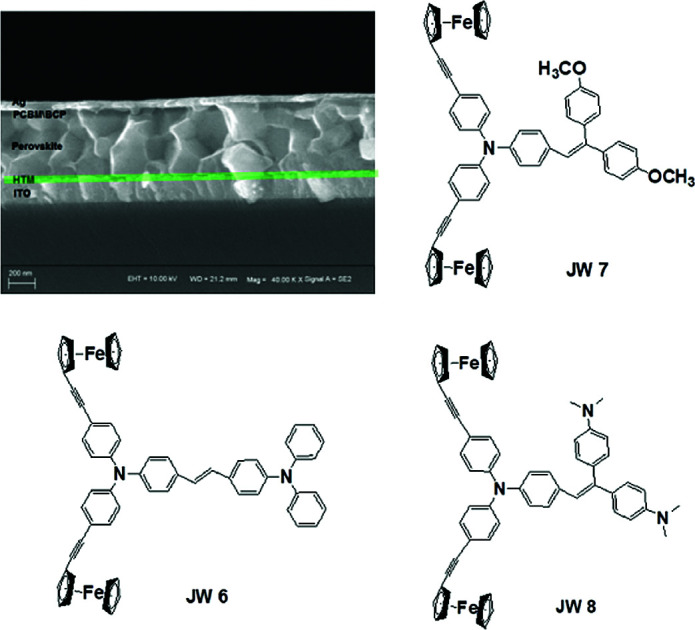

## 1 Introduction

Perovskite solar cells have attracted much attention due to the high photovoltaic performance of the organolead halide.^1–7^ The organolead halide perovskite was used as the light absorber in liquid-based dye-sensitized solar cells (DSSCs), and showed a power conversion efficiency (PCE) of 3.8% in 2009. The initial PCE of the solid-state perovskite solar cells was 9.7% in 2012.^8,9^ Recently, PCE of perovskite solar cells has been over 22%.^10^ The commonly reported CH_3_NH_3_PbI_3_ was used in the first solid-state PSC, and a solid hole-transport layer was made of 2,20,7,70-tetrakis-(*N*,*N*-di-*p*-methoxyphenylamine)-9,90-spirobifluorene (spiro-OMeTAD), which shows good charge extraction and improves the long-term stability of the device.^9,11–15^ It is necessary to place the perovskite between an n-type semiconductor layer and a p-type semiconductor layer, according to the structure of traditional PSCs,^16^ for charge separation and transport in a PSC device. Organic small-molecule HTMs such as spiro-OMeTAD are commonly used as p-type semiconductor materials for PSCs. Compared with inorganic HTMs and organic polymer HTMs, organic small-molecule HTMs have the advantages of facile synthesis, flexible modification of structure, and easy purification.^17–22^ Organic small-molecule HTMs can reach high hole mobility by possessing tunable energy levels, suitable solubility in organic solvents, and good film-forming properties.^23–26^

Ferrocene is an organometallic compound with a sandwich structure. Ferrocene and its derivatives are widely used in electrochemical sensors, biomedicine, chemical catalysts and photovoltaic materials due to their good redox abilities.^27–30^ Ferrocene can also act as a suitable electron-donating unit and can be easily functionalized, due to which it has been reacted with small molecules for conjugation. Ferrocene-containing triphenylamine derivatives have been reported because of their good light-harvesting ability and fast charge regeneration.^31,32^ Ferrocenyl-substituted triphenylamines are used as sensitizers in DCCCs, and an inserted π-linker between ferrocene and triphenylamine is beneficial for charge transport in the molecule.^33–35^ Structural changes have a significant influence on electron delocalization though the entire molecule. Ferrocenyl-substituted triphenylamines have also been used as p-type redox promoters in electrochromic display devices, and they can form a film layer by spin-coating.^36^ Therefore, ferrocene is a promising candidate for photovoltaic materials. In our research, new ferrocenyl-substituted triphenylamine derivatives were synthesized, and they were used as organic small-molecule HTMs to construct PSCs. To the best of our knowledge, this is the first time that organic small molecules containing ferrocene have been reported in PSCs.

**Scheme 1:**
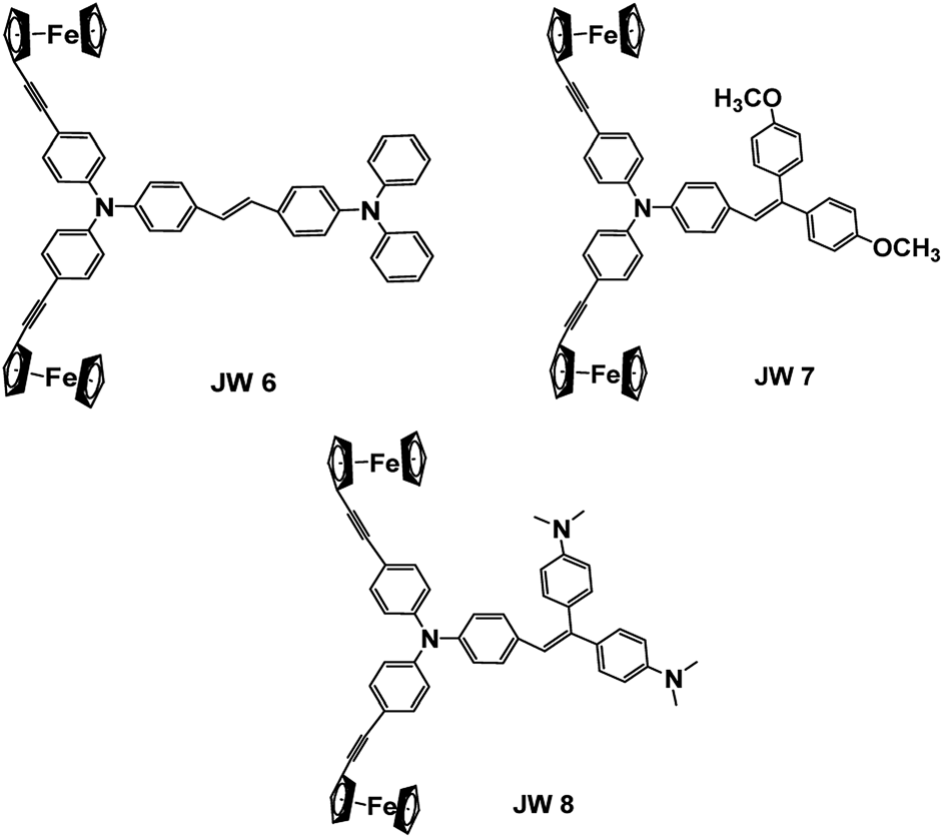
Chemical structures of JW6, JW7 and JW8.

In this study, three new ferrocene-containing compounds (JW6, JW7 and JW8) were prepared, and their structures are shown in Scheme 1. In JW6, JW7 and JW8 molecules, biferrocene groups were connected with triphenylamines *via* triple bonds, and a double bond was also introduced to link a moiety of triphenylamine, bis(4-methoxyphenyl)methane and 4,4′-methylenebis(*N*,*N*-dimethylaniline), which can adjust the solubility, energy levels and charge transport. Theoretical calculations were performed to observe the optimized geometric structures and frontier molecular orbitals of the three compounds. Spectral and electrochemical measurements were applied for the determination of light absorption and energy levels. Differential scanning calorimetry (DSC) was performed for analysing the thermal stability of JW6, JW7 and JW8. PSCs based on JW6, JW7 and JW8 were fabricated, and we found that the concentration of HTM solution significantly influenced the device performance. In the inverted planar heterojunction perovskite solar cells (p-i-n), the concentration of HTMs was 5 mg mL^−1^ without any dopant. The PCE of p-i-n cells based on the studied HTMs reached 89% of the PCE of those cells based on the standard HTM (poly(3,4-ethylenedioxythiophene), PEDOT). In devices of the regular mesoporous TiO_2_-based PSCs (n-i-p), different concentrations of HTM solutions were spin-coated by adding only 4-*tert*-butylpyridine (TBP). A low concentration of 10 mg mL^−1^ was good for the studied HTM to establish excellent contact with the perovskite layer. Among the three compounds, JW7 showed better photovoltaic performances than JW6 and JW8. The structure–efficiency relationship of these ferrocene-containing HTMs was discussed based on the measurements of *J*–*V* characterizations and steady-state and time-resolved photoluminescence (PL) spectra. The hole mobility of the new HTMs was measured by space-charge-limited-current (SCLC), and charge recombination was studied by electrochemical impedance spectroscopy (EIS). Through this research we hope to provide some experimental basis for designing new HTMs for PSCs.

## 2 Experimental

### 2.1 Materials

All the starting materials used in this study were purchased from J&K Chemical, Energy Chemical or Sigma-Aldrich and used without further purification, unless otherwise stated. *N*,*N*-Dimethylformamide (DMF) and tetrahydrofuran (THF) were dried using 200 mesh molecular sieve and sodium metal, respectively.

### 2.2 Fabrication of PSCs

In this study, two architectures of PSCs were fabricated. The conventional n-i-p type PSCs (FTO/TiO_2_/perovskite/HTM/Ag) were fabricated according to the published method.^37^ The main study was focussed on the inverted p-i-n type of PSCs (ITO/HTM/perovskite/PCBM/BCP/Ag). In brief, ITO glasses were successively washed using a special cleaner, acetone, water and ethanol, and then covered with HTM layer (5 mg mL^−1^) by spinning at 4000 rpm for 40 s. This first layer was sintered at 100 °C for 10 min. By the sequential deposition method, a perovskite layer was covered on the HTM layer. After the perovskite precursor solution (461 mg PbI_2_, 159 mg MAI, 70.9 μL DMSO and 634.92 μL DMF) was spin-coated at 5000 rpm for 25 s in a nitrogen filled glovebox, the device was annealed at 100 °C for 10 min. Then, PCBM (20 mg mL^−1^) in CB and BCP (0.5 mg mL^−1^) in isopropanol were spin-coated on the perovskite layer at 4000 rpm for 40 s. The PCBM layer was annealed at 80 °C for 10 min, while BCP was only spin-coated on the PCBM layer without any heating. Finally, Ag was vacuum-evaporated on the top of the device as the counter electrode.

### 2.3 Characterizations

^1^H NMR and ^13^C NMR spectra were recorded on a Bruker AM-400 spectrometer. The chemical shifts were reported against TMS. High resolution mass spectra were obtained on a Micromass GCT-TOF mass spectrometer. UV-vis absorption spectra were recorded on a SHIMADZU UV-2600 spectrophotometer. Photoluminescence (PL) measurements were recorded on a HITACHI F-4500 fluorescence spectrophotometer at 500 nm excitation wavelength. Cyclic voltammetry (CV) measurements were recorded using a Zennium electrochemical workstation (ZAHNER, Germany) under a three-electrode system, platinum was used as the working electrode, Ag/AgCl electrode was used as the reference electrode, and Pt-wires were used as the counter electrode. Electrochemical impedance spectroscopy (EIS) in the frequency range from 200 mHz to 100 kHz was performed with a Zennium electrochemical workstation (ZAHNER, Germany) in the dark with the alternate current amplitude set at 10 mV. Current–voltage (*J*–*V*) characteristics of PSCs were measured using a Keithley 2400 digital SourceMeter controlled by a computer under a standard AM 1.5 solar simulator (Oriel 91160-1000 (300 W) Solar Simulator). Incident photon-to-current conversion efficiency (IPCE) for PSCs was recorded on the QTest Station 2000 IPCE measurement system, CROWNTECH, USA. The space-charge-limited-current (SCLC) method was used to measure the hole mobility of HTMs using the equation:^37^
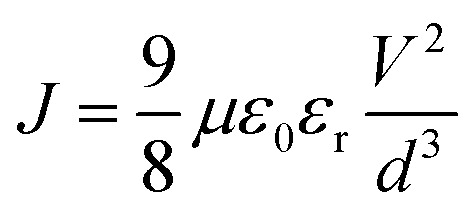


### 2.4 Synthesis of JW6, JW7 and JW8

**Scheme 2:**
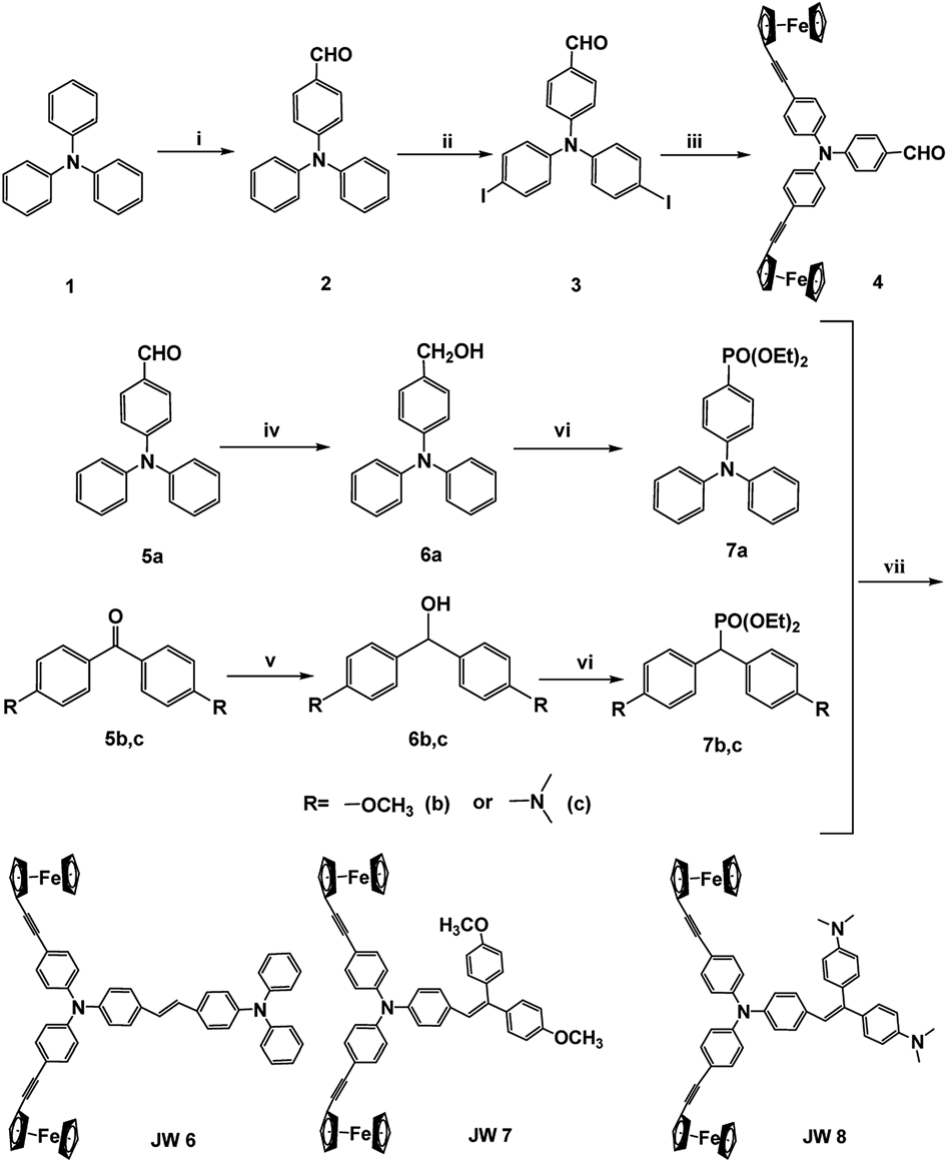
The synthesis routes for JW6, JW7 and JW8. Conditions: (i) POCl_3_, DMF, 0 °C, 0.5 h then RT, 5 h; (ii) NIS, CHCl_3_/AcOH(4 : 1), 100 °C, 0.5 h; (iii) ethynyl ferrocene, Pd(PPh_3_)_4_, Et_3_N/THF (1 : 1), N_2_, reflux, 6 h; (iv) NaBH_4_, EtOH, NaOH, N_2_, 0 °C, 0.5 h then RT, 4 h; (v) NaBH_4_, i-PrOH, H_2_O, N_2_, reflux, 6 h; (vi) P(OEt)_3_, I_2_, RT, 5 h; (vii) 4, *t*-BuOK, THF, RT, 10 h.

The synthesis routes for JW6, JW7 and JW8 are shown in Scheme 2. The intermediates of compounds 1–7 have been reported in literature reports.^34,38–41^

#### General procedure for the synthesis of compounds JW6, JW7 and JW8

Compounds 7a–c (0.677 mmol) were dissolved using anhydrous THF in a 100 mL two-neck round-bottom flask under N_2_. After the temperature dropped to 0 °C, the THF solution of *t*-BuOK (0.880 mmol) was added dropwise, and the solution turned yellow. The reaction was kept at 0 °C for 1 hour. Then, THF solution of 4 (0.564 mmol) was added slowly. The mixture was then placed at room temperature and stirred overnight. Then, saturated NH_4_Cl was added to stop the reaction and the product was extracted with dichloromethane. The combined organic layer was washed with brine and dried over anhydrous Na_2_SO_4_. The solvent was evaporated, and the remaining crude product was purified by column chromatography to obtain the desired compounds JW6, JW7 and JW8.

Characterization details for the synthesis of JW6, JW7 and JW8 are provided in ESI.†

## 3 Results and discussion

### 3.1 Spectral, electrochemical and thermal stability measurements

**Fig. 1:**
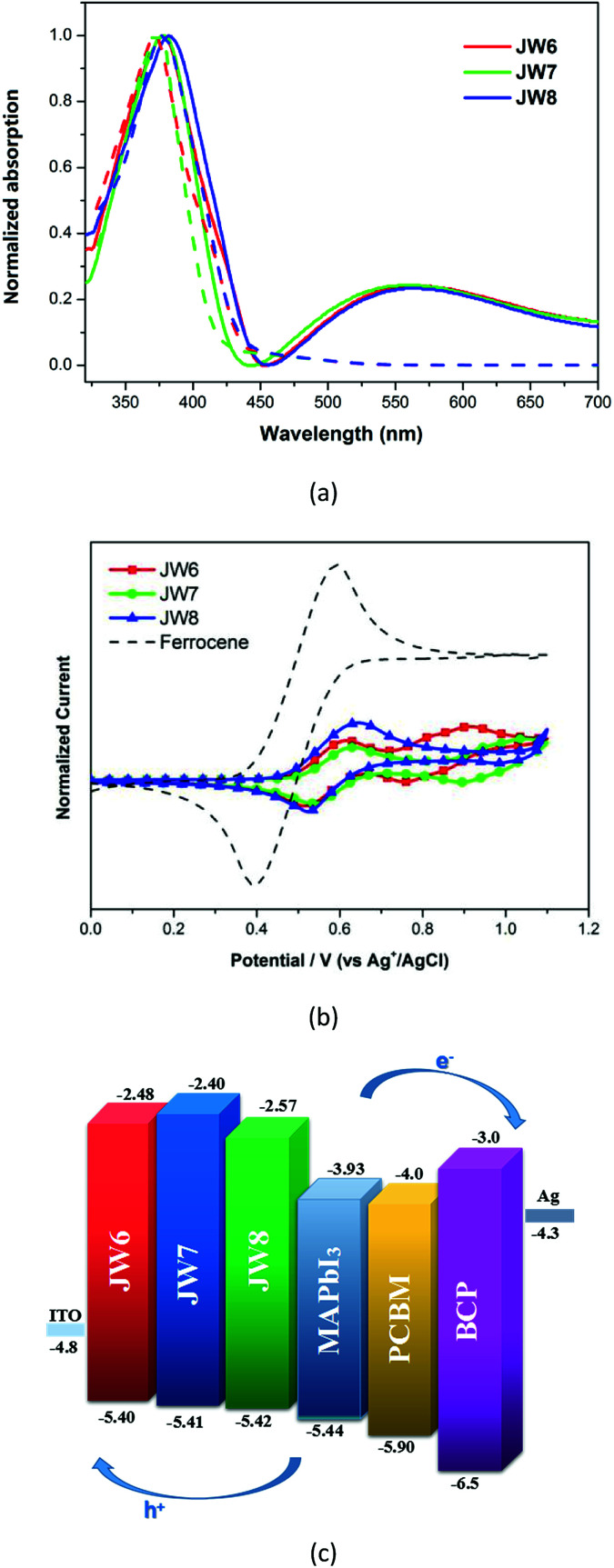
(a) Absorption spectra of JW6, JW7 and JW8 in film (solid line) and dichloromethane solution (dash line); (b) cyclic voltammetry of JW6, JW7 and JW8; (c) energy level scheme of JW6, JW7 and JW8 in a p-i-n type PSC.

Suitable HOMO and LUMO levels are important for HTMs to achieve hole transport in a PSC device. The HOMO levels matching the valence band energy of CH_3_NH_3_PbI_3_ are suitable for fabricating efficient PSCs. In this study, the energy levels of the three HTMs were determined by spectroscopic and electrochemical measurements. As shown in Fig. 1(a), UV-visible absorptions of JW6, JW7 and JW8 were measured in film and in dichloromethane solution. The energy gap (*E*_0–0_) values of JW6, JW7 and JW8 were calculated from the onset wavelengths of the absorption spectra in solution, which were 2.92 eV, 3.01 eV and 2.85 eV, respectively. JW8 showed the smallest *E*_0–0_ value among the three HTMs, suggesting that JW8 possesses larger π-conjugation than JW6 and JW7. This was consistent with the maximum absorption peak of JW8 displaying a red shift in comparison with that of JW6 and JW7 both in dichloromethane solution and in solid-state film. The absorption of the studied HTMs bearing biferrocene was extended to around 450 nm. Cyclic voltammetry (CV) was used to measure the oxidation and reduction peaks of JW6, JW7 and JW8 in dichloromethane solution, and the CV curves are shown in Fig. 1(b). The HOMO levels of JW6, JW7 and JW8 were calculated from the equation *E*_HOMO_ = −4.7 − *E*_ox_. The corresponding *E*_HOMO_ values of JW6, JW7 and JW8 were −5.40 eV, −5.41 eV and −5.42 eV, respectively. The *E*_HOMO_ values of the studied HTMs were very close and slightly higher than the valence band energy of CH_3_NH_3_PbI_3_. Thus, it was easy for the new HTMs to accept holes transferred from CH_3_NH_3_PbI_3_. The LUMO levels of JW6, JW7 and JW8 were calculated from the equation *E*_LUMO_ = *E*_HOMO_ + *E*_0–0_, and the corresponding *E*_LUMO_ values were −2.48 eV, −2.40 eV and −2.57 eV, respectively. The higher *E*_LUMO_ value of JW7 can more effectively prevent the electron transfer from CH_3_NH_3_PbI_3_ to HTMs, and thus reduce charge recombination in the device. Moreover, the energy level scheme for a p-i-n cell is represented in Fig. 1(c), in which the difference in HOMO and LUMO energy levels of the three compounds can be clearly seen. The related spectral and electrochemical results are listed in Table 1.

**Table 1:** Spectral and electrochemical properties of JW6, JW7 and JW8

HTMs	*λ* ^sol^_max_ a/nm	*λ* ^film^_max_ b/nm	*λ*_onset_/nm	Mobility/cm^2^ V^−1^ s^−1^	*E*_0–0_ c/eV	*E*_HOMO_ d/eV	*E*_LUMO_ e/eV
JW6	372	378	425	1.3 × 10^−4^	2.92	−5.40	−2.48
JW7	372	378	412	4.3 × 10^−4^	3.01	−5.41	−2.40
JW8	378	382	435	1.5 × 10^−4^	2.85	−5.42	−2.57

a UV-vis spectra were measured in dichloromethane solution (5 × 10^−6^ M).

b *λ*_onset_ is the onset wavelength of absorption spectrum.

c Optical band gap was calculated from 1240/*λ*_onset_.

d *E*_HOMO_ was calculated from the equation *E*_HOMO_ = −4.7 − *E*_ox_. *E*_ox_ was standardized with ferrocene (0.63 V *vs.* NHE).

e *E*_LUMO_ = *E*_HOMO_ + *E*_0–0_.

**Fig. 2:**
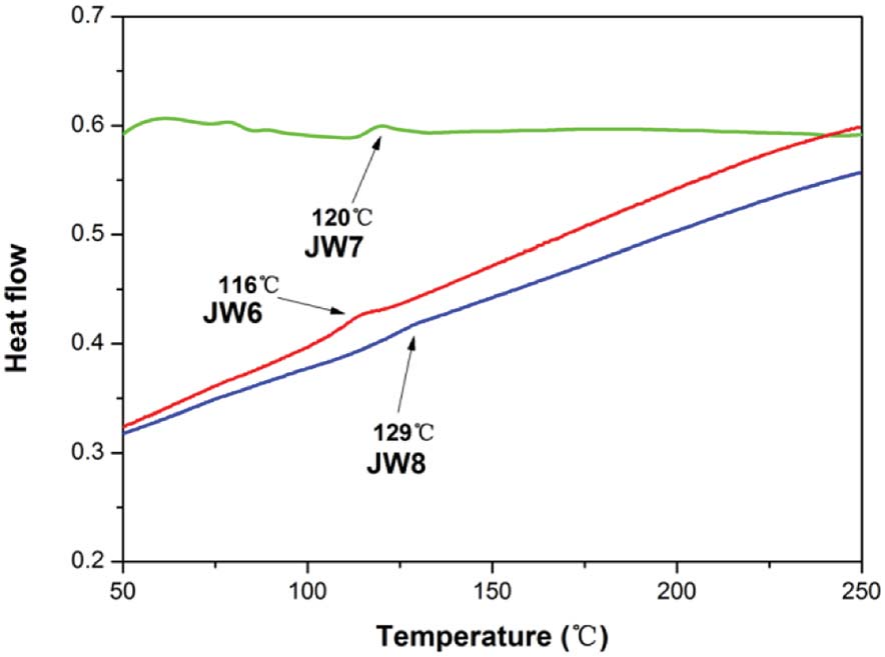
DSC curves of JW6, JW7 and JW8.

Differential scanning calorimetry (DSC) was used to test the glass transition temperature (*T*_g_) of JW6, JW7 and JW8. DSC curves of these new HTMs are displayed in Fig. 2. JW8 possessing 4,4′-methylenebis(*N*,*N*-dimethylaniline) and JW7 possessing bis(4-methoxyphenyl)methane have higher *T*_g_ values (129 °C and 120 °C) than JW6 possessing triphenylamine (116 °C). The DCS result shows JW6, JW7 and JW8 to have good stability in the amorphous state.

### 3.2 Theoretical calculations

**Fig. 3:**
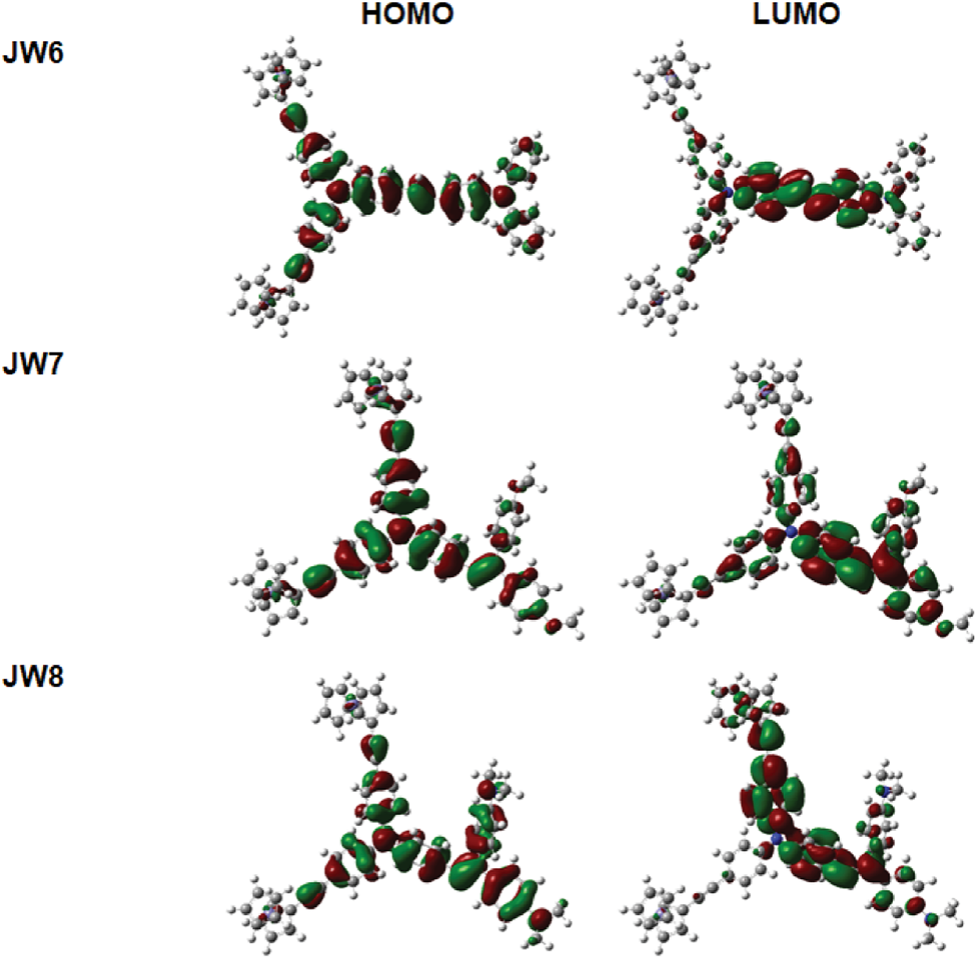
Frontier molecular orbitals of JW6, JW7 and JW8.

The substituents have an important influence on the electron distribution and conjugated construction of HTM molecules. Theoretical calculations on the basis of DFT at the B3LYP/6-31G(g,d) level were used to optimize the geometric structures of the new HTMs. The optimized structures of JW6, JW7 and JW8 are given in Fig. S1,† and the representative bond lengths and dihedral angles are listed in Table S1 (seen in ESI†). The frontier molecular orbitals of the three compounds are shown in Fig. 3. The highest occupied molecular orbitals (HOMO) of the three compounds are almost delocalized over the whole molecule. The lowest unoccupied molecular orbitals (LUMO) are localized in the double bond part. The LUMO of JW7 is transferred from the triphenylamine part to the (4-methoxyphenyl)methane section. As shown in Table S1,† the C–C bond lengths of ferrocenylethynyl and triphenylamine in the three compounds are the same (1.423 Å), and the C–N bond lengths of this triphenylamine are similar, which are 1.419 Å, 1.418 Å and 1.416 Å for JW6, JW7 and JW8, respectively. JW7 and JW8 have a shorter C–N bond length, suggesting a longer π-conjugated length. Moreover, JW7 and JW8 have similar C=C bond lengths of 1.468 Å and 1.467 Å, respectively. The C–C bonds of double bond and diphenylmethane group for JW7 and JW8 are 1.491 Å and 1.489 Å, respectively. Overall, JW8 has the shortest bond length among the three HTMs. The dihedral angles of the two phenyl rings of the triphenylamine connecting ferrocenylethynyl in JW6, JW7 and JW8 are 40.9°, 39.4° and 37.9°, respectively. The dihedral angles between the double bond and the phenyl ring of diphenylmethane group in JW7 and JW8 are −26.9° and −25.2°, respectively. The data of dihedral angles also indicate that JW7 and JW8 have a more planar conjugated plane than JW6. Therefore, the results suggest that JW7 and JW8 may have a trend for faster charge transport than JW6. The device performances of the three compounds will be discussed in detail in Section 3.3.

### 3.3 Device performance

**Fig. 4:**
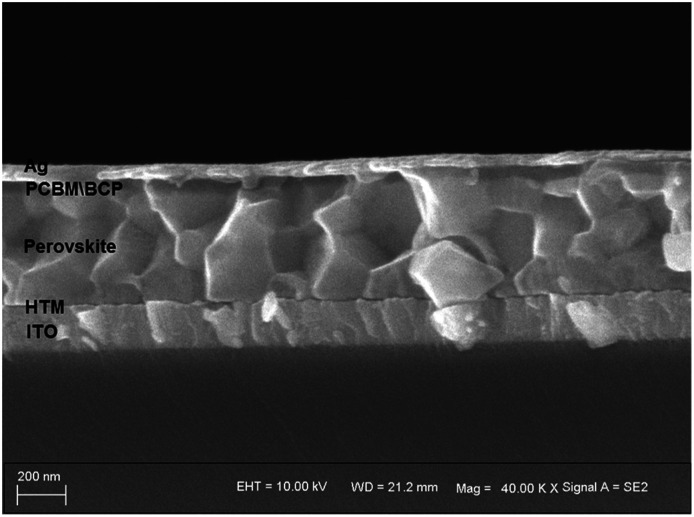
Cross-section SEM image of the p-i-n type cell fabricated using JW7.

**Fig. 5:**
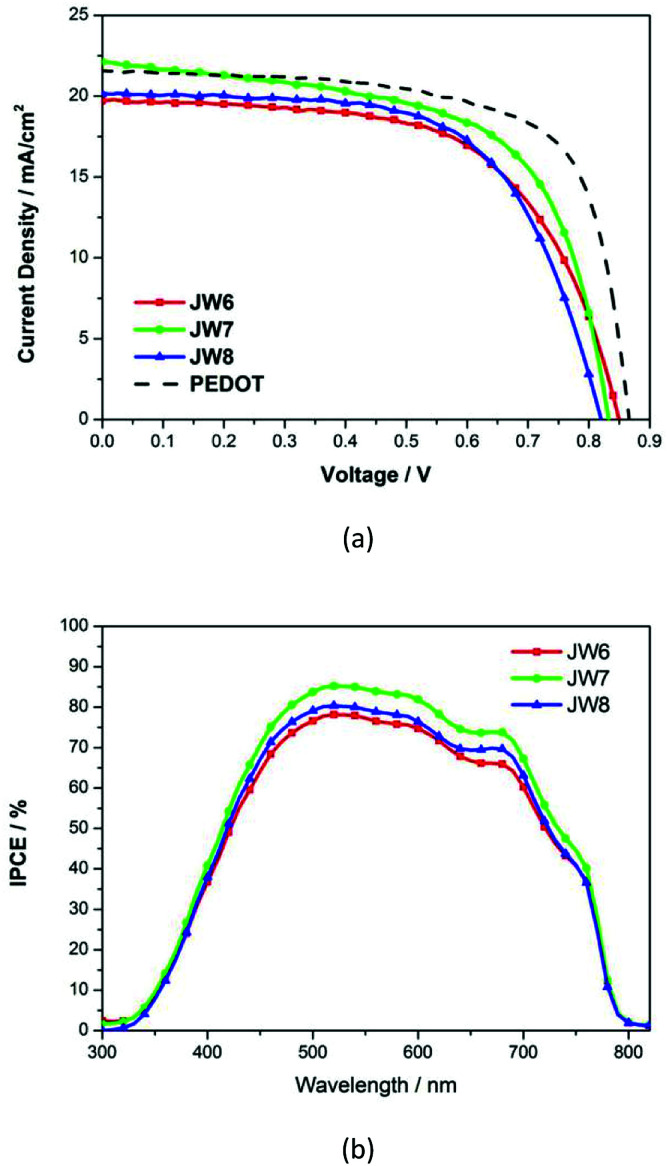
(a) *J–V* characteristic curves and (b) IPCE spectra for p-i-n PSCs.

HTM solutions were prepared to be spin-coated for fabricating PSC devices, and two architectures of PSCs were constructed to evaluate the photovoltaic performances of the new HTMs-based cells. In the inverted planar heterojunction perovskite solar cells (p-i-n), the studied HTMs are used without any additives. The concentration of HTM solutions was 5 mg mL^−1^. The p-i-n cells were based on the structure of ITO/HTM/perovskite/PCBM/BCP/Ag. Fig. 4 shows a cross-section SEM image of the p-i-n-type cell. The structure of the device is dense and neat. The fill factor (FF) of p-i-n cells fabricated by using JW6, JW7 and JW8 is higher than that of their n-i-p cells. The PCE of p-i-n cells based on JW6, JW7 and JW8 are 10.23%, 11.43% and 10.42%, respectively. The PCE of PSCs fabricated using the standard PEDOT is 12.86%. The corresponding *J*–*V* curves of p-i-n cells are shown in Fig. 5(a). In addition, device performances of p-i-n cells display replicability and stability. The photovoltaic parameters of eight cells of p-i-n-type PSCs fabricated using JW7 are presented in Table S2.† They are kept at room temperature (25 °C) and in ambient air of 30% RH without encapsulation. Hysteresis test for p-i-n type PSCs based on the new HTMs was conducted, and the results are summarized in Table S3.† Fig. S2† exhibits the *J*–*V* plots of p-i-n type PSCs based on JW7 through reverse and forward bias at a scan rate of 0.1 V s^−1^. The data of hysteresis test for p-i-n type PSCs fabricated using the standard PEDOT are also provided in Table S3† as a reference.

Results of the incident photon-to-current conversion efficiency (IPCE) spectra of p-i-n cells fabricated using JW6, JW7 and JW8 are shown in Fig. 5(b). The three HTMs have a wide range from 450 nm to 700 nm. The difference in *J*_SC_ is in accordance with that in the measured values in the order of JW7 > JW8 > JW6 (Table 2).

**Table 2:** *J*–*V* parameters of PSCs (p-i-n type) with JW6, JW7 and JW8

HTMs	*V*_OC_/mV	*J*_SC_/mA cm^−2^	FF	PCE/%
JW6	850	19.73	0.61	10.23
JW7	833	22.13	0.62	11.43
JW8	820	20.17	0.63	10.42
PEDOT	867	22.26	0.69	12.86

**Fig. 6:**
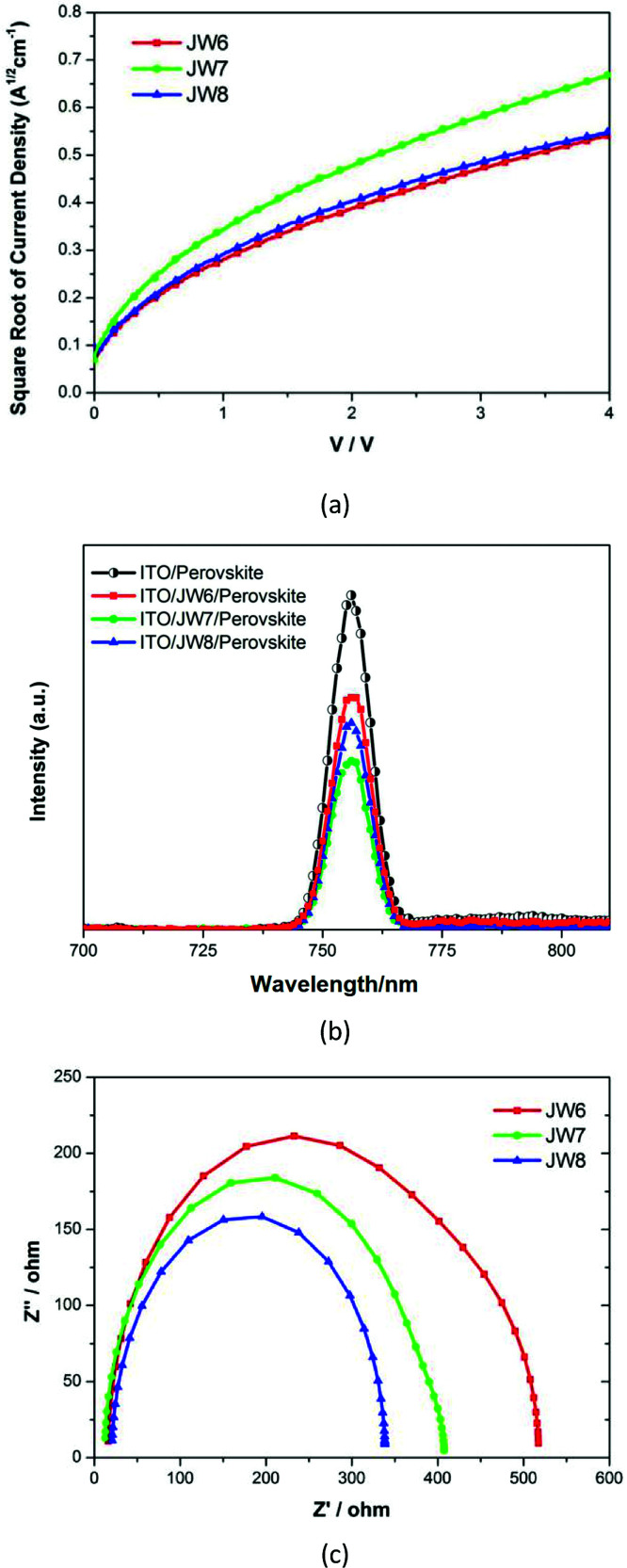
(a) The space-charge-limited-current (SCLC) measurements of hole-only devices of ITO/HTM/PCBM/BCP/Ag based on JW6, JW7 and JW8. (b) Steady-state and time-resolved photoluminescence (PL) spectra of JW6, JW7 and JW8. (c) EIS for PSCs based on JW6, JW7 and JW8 measured in the dark under 0.9 V bias displayed in the form of Nyquist plots.

Furthermore, data based on the space-charge-limited-current (SCLC) method was used to study the hole mobility of JW6, JW7 and JW8. Fig. 6(a) shows the fitting current density–voltage (*J*–*V*) curves. The calculated values of the hole mobility of JW6, JW7 and JW8 were 1.3 × 10^−4^ cm^2^ V^−1^ s^−1^, 4.2 × 10^−4^ cm^2^ V^−1^ s^−1^ and 1.5 × 10^−4^ cm^2^ V^−1^ s^−1^. JW7 showed higher hole mobility than JW6 and JW8, indicating that different substitutes had significant influence on the intermolecular charge transport of the studied HTMs. Photoluminescence (PL) measurements were recorded to study the charge extraction capability of JW6, JW7 and JW8, and those for PEDOT were also recorded as a standard. Fig. 6(b) displays the steady-state PL spectra of the CH_3_NH_3_PbI_3_ perovskite films without or with capping different HTMs. The PL band of the perovskite film is observed to be centered at 756 nm by excitation at 500 nm. All the bilayers show a drastic quenching of PL with respect to the pristine perovskite, following the order of perovskite/JW7 > perovskite/JW8 > perovskite/JW6. This suggests that JW7 has a more positive effect on device performances than JW6 and JW8, benefiting from the higher hole mobility and hole extraction capability.^42^ Electrochemical impedance spectroscopy (EIS) was performed to further understand charge recombination in PSCs fabricated using JW6, JW7 and JW8. Fig. 6(c) displays the Nyquist plots. The recombination resistance (*R*_rec_) is represented by the large arc at low frequency. The *R*_rec_ value increases in the sequence of JW8, JW7 and JW6, indicating that the device performances based on JW8 are affected by faster charge recombination. Larger *R*_rec_ of JW6 and JW7 are beneficial for reducing charge recombination.^37^ However, the device performances based on JW6 are limited by relatively lower hole mobility, according to data of PL measurements. Therefore, JW7 with the 4,4′-methylenebis(*N*,*N*-dimethylaniline) substituent shows a good performance in the inverted planar heterojunction perovskite solar cells.

**Fig. 7:**
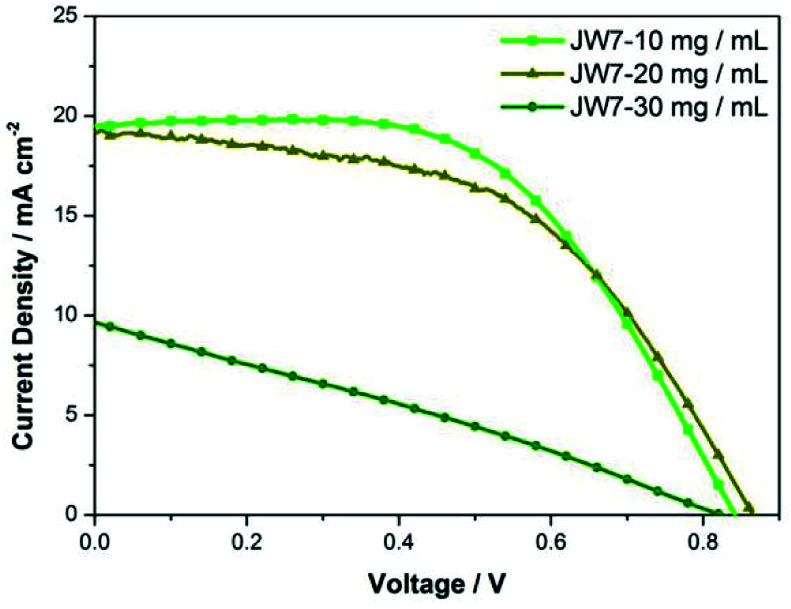
*J*–*V* characteristic curves for PSCs (n-i-p type).

In addition, different concentrations of JW7 were tested in the regular mesoporous TiO_2_-based perovskite solar cells (n-i-p). The structure of the n-i-p-type cells is FTO/TiO_2_/perovskite/HTM/Ag. The photovoltaic parameters of the representative devices based on n-i-p-type PSCs are listed in Table 3. All the n-i-p cells are based on HTM solutions prepared by adding only TBP and without adding expensive additives such as LiTFSI. Fig. 7 shows the effect of different concentrations on n-i-p device performances determined by *J*–*V* measurements. When using JW7 of 30 mg mL^−1^, the value of photocurrent (*J*_SC_) drops sharply, and the highest PCE is only 2.23%. The PCE increases to 8.65% when the concentration of JW7 is reduced to 20 mg mL^−1^. Furthermore, the device performances get even better when the concentration of HTM solution decreases to 10 mg mL^−1^, and the PCE can reach 9.36%.

**Table 3:** *J*–*V* parameters of n-i-p type PSCs with different concentrations of JW7

Conditions	*V*_OC_/mV	*J*_SC_/mA cm^−2^	FF	PCE/%
1^a^	825	9.64	0.28	2.23
2^a^	881	8.87	0.26	2.03
3^b^	865	19.24	0.52	8.65
4^b^	836	20.92	0.47	8.22
5^b^	870	18.99	0.47	7.77
6^b^	864	18.95	0.46	7.53
7^c^	842	19.50	0.57	9.36
8^c^	825	19.21	0.56	8.88
9^c^	847	18.45	0.53	8.28

a PSC covered with 30 mg mL^−1^ HTM.

b PSC covered with 20 mg mL^−1^ HTM.

c PSC covered with 10 mg mL^−1^ HTM.

## 4 Conclusions

In this study, three triphenylamine derivatives containing ferrocene were synthesized and used as organic HTMs for fabricating efficient PSCs. The substitutes on their structures are different, which are triphenylamine for JW6, bis(4-methoxyphenyl)methane for JW7 and 4,4′-methylenebis(*N*,*N*-dimethylaniline) for JW8. The maximum absorption wavelengths of the three compounds are around 370–380 nm and that of JW8 (378 nm) has a red shift when compared with JW6 (372 nm) and JW7 (372 nm). Theoretical calculations show that JW7 and JW8 have a more planar conjugated structure. The three HTMs display *T*_g_ values beyond 110 °C. SCLC results show that JW7 exhibits the highest hole mobility among the studied HTMs. In the inverted p-i-n-type of PSCs (ITO/HTM/perovskite/PCBM/BCP/Ag), JW7 shows better photovoltaic performance than JW6 and JW8 under the same conditions, leading to a PCE of 11.43% (standard PEDOT, 12.86%). In the conventional n-i-p device of FTO/TiO_2_/perovskite/HTM/Ag, the optimal concentration of HTM is 10 mg mL^−1^. The corresponding highest PCE for these n-i-p cells was 9.36%, which were fabricated using JW7 and adding TBP as the dopant. Therefore, ferrocene-containing materials have a great potential in perovskite solar cells, and there is a lot of work to be done in the development of these ferrocene-based HTMs for fabricating high-efficiency PSCs.

## Conflicts of interest

There are no conflicts to declare.

## Supplementary Material

RA-009-C8RA08946A-s001
