# Supplementary material for: New ferrocenyl-containing organic hole-transporting materials for perovskite solar cells in regular (n-i-p) and inverted (p-i-n) architectures
Source: RSC Adv. 2019 Jan 2;9(1):216–23. doi: 10.1039/c8ra08946a (PMC9059360; doi:10.1039/c8ra08946a)
Supplement: RA-009-C8RA08946A-s001 [file RA-009-C8RA08946A-s001.pdf]

Electronic Supplementary Material (ESI) for RSC Advances.
This journal is © The Royal Society of Chemistry 2018

*Supporting Information*

# New ferrocenyl-containing organic hole-transporting materials for perovskite solar cells in regular (n-i-p) and inverted (p-i-n) architectures

Jingwen Jia, Liangsheng Duan, Yu Chen*, Xueping Zong, Zhe Sun, Quanping Wu, Song Xue*

*Tianjin Key Laboratory of Organic Solar Cells and Photochemical Conversion, School of Chemistry & Chemical Engineering, Tianjin University of Technology, Tianjin 300384, PR China*

**Corresponding authors. E-mail addresses: cytjut@163.com (Y. Chen), xuesong@ustc.edu.cn (S. Xue).*

Table of Contents

Characterization details for the synthesis of **JW6**, **JW7** and **JW8** …………………S3

The optimized structures of **JW6**, **JW7** and **JW8** …………………………………S4

Representative bond lengths and dihedral angles of optimized structures of **JW6**, **JW7** and **JW8**..……………………………………………………………...S5

The photovoltaic parameters from eight cells of p-i-n type PSCs fabricated by **JW7**……………………………………………………………………… .S6

The photovoltaic parameters of hysteresis test for p-i-n type PSCs with various HTMs..……………………………………………………………………..S7

J-V plots of p-i-n type PSCs based on **JW7** measured through reverse and forward bias at a scan rate of 0.1V/s. ……………………………………………………..….S8

$^{1}$H NMR and $^{13}$C NMR spectra of **JW6**, **JW7** and **JW8**...…………………..… S9-S11

**Characterization details for the synthesis of JW6, JW7 and JW8**

**JW6**, orange solid (63.2% yield). $^{1}$H NMR (400 MHz, $CDCl_3$) δ 7.44-7.39 (m, 8H), 7.30 (t, *J* = 7.8 Hz, 4H), 7.15 (d, *J* = 7.8 Hz, 4H), 7.12 (s, 2H), 7.08 (d, *J* = 8.6 Hz, 8H), 7.02-6.98 (m, 2H), 4.53 (s, 4H), 4.28 (s, 14H). $^{13}$C NMR (101 MHz, $CDCl_3$) δ 147.58, 146.55, 132.45, 129.92, 129.69, 129.28, 127.24, 124.57, 124.17, 123.72, 123.02, 122.91, 118.12, 87.82, 85.66, 71.34, 69.96, 68.74, 65.59. HRMS (ESI) (m/z): $[M]^+$ calcd. For $C_{62}H_{46}N_2Fe_2$, 931.2360; found: 931.2441.

**JW7**, orange solid (51.0% yield). $^{1}$H NMR (400 MHz, $CDCl_3$) δ 7.41-7.33 (m, 6H), 7.29 (d, *J* = 8.5 Hz, 4H), 7.18 (d, *J* = 8.5 Hz, 2H), 7.03 (d, *J* = 8.6 Hz, 4H), 6.92 (d, *J* = 8.7 Hz, 2H), 6.89 (d, *J* = 6.3 Hz, 2H), 6.87 (d, *J* = 6.5 Hz, 2H), 4.51 (s, 4H), 4.27 (s, 14H), 3.87 (s, 3H), 3.84 (s, 3H). $^{13}$C NMR (100 MHz, $CDCl_3$) δ 159.22, 146.53, 144.96, 136.47, 132.40, 131.42, 130.35, 128.65, 125.48, 123.9, 123.72, 118.16, 114.17, 113.62, 87.80, 85.69, 71.34, 69.95, 68.71, 65.66, 55.23. HRMS (ESI) (m/z): $[M]^+$ calcd. For $C_{58}H_{46}NO_2Fe_2$, 900.4129; found: 900.4230.

**JW8**, orange solid (59.3% yield). $^{1}$H NMR (400 MHz, $CDCl_3$) δ7.42-7.37 (m, 6H), 7.26 (d, *J* = 8.5 Hz, 2H), 7.13 (d, *J* = 8.5 Hz, 2H), 7.02 (d, *J* = 8.4 Hz, 4H), 7.00 (d, *J* = 8.5 Hz, 2H), 6.88 (d, *J* = 8.4 Hz, 2H), 6.74-6.69 (m, 3H), 4.51 (s, 4H), 4.26 (s, 14H), 3.01 (s, 6H), 2.99 (s, 6H). $^{13}$C NMR (100 MHz, $CDCl_3$) δ 146.61, 144.23, 142.04, 132.34, 131.14, 130.93, 130.15, 128.86, 128.52, 124.17, 123.53, 117.77, 112.38, 112.03, 87.63, 85.73, 71.32, 69.95, 68.71, 65.65, 40.50. HRMS (ESI) (m/z): $[M]^+$ calcd. For $C_{60}H_{51}N_3Fe_2$, 926.2782; found: 926.2863.

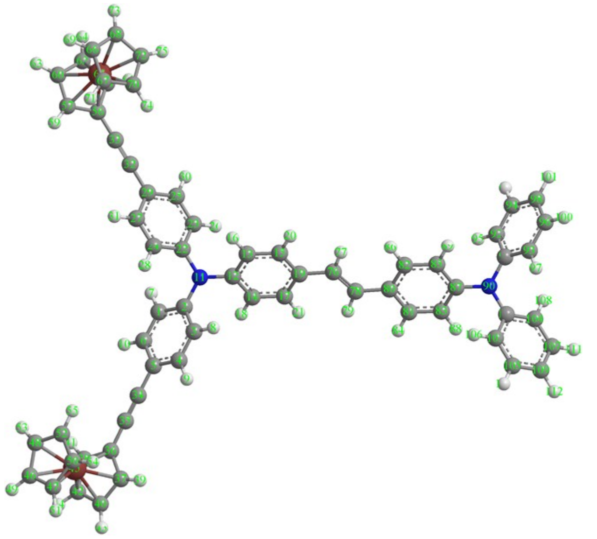

**JW6**

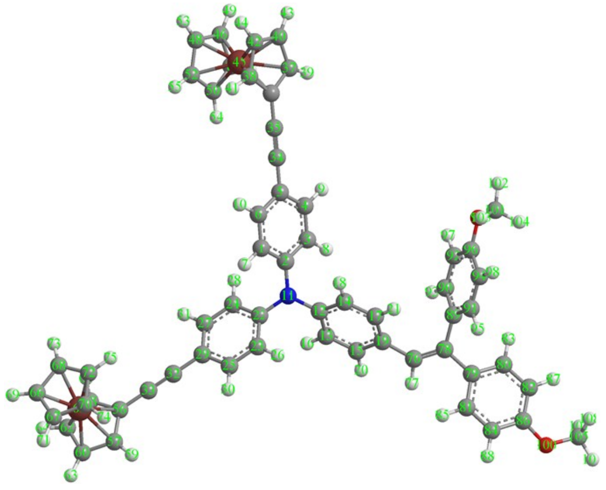

**JW7**

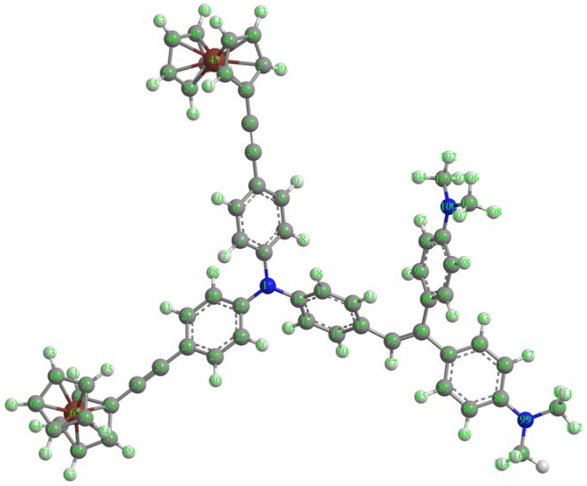

**JW8**

**Fig.S1**. The optimized structures of **JW6**, **JW7** and **JW8**.

**Table S1**. Representative bond lengths and dihedral angles of optimized structures of **JW6**, **JW7** and **JW8**.

| Compounds | Bond lengths (Å) | | dihedral angles (°) | |
|---|---|---|---|---|
| **JW6** | C(32)-C(29) | 1.423 | C(23)-C(22)-N(11)-C(12) | 40.9 |
| | C(22) -N(11) | 1.419 | C(78)-C(76)-C(19)C(17) | -5.1 |
| | C(76)-C(19) | 1.462 | C(91)-N(90)-C(87)-C(85) | -39.2 |
| | N(90)-C(87) | 1.417 | | |
| **JW7** | C(34)-C(5) | 1.423 | C(78)-C(76)-C(19)C(17) | -26.9 |
| | N(11)-C(2) | 1.418 | C(12)-N(11)-C(2)-C(3) | 39.4 |
| | C(76)-C(19) | 1.468 | | |
| | C(89)-C(78) | 1.491 | | |
| **JW8** | C(34)-C(5) | 1.423 | C(78)-C(76)-C(19)C(17) | -25.2 |
| | N(11)-C(2) | 1.416 | C(12)-N(11)-C(2)-C(3) | 37.9 |
| | C(76)-C(19) | 1.467 | | |
| | C(89)-C(78) | 1.489 | | |

**Table S2.** The photovoltaic parameters from eight cells of p-i-n type PSCs fabricated by **JW7**.

| **Cell** | $V_{OC}$ / mV | $J_{SC}$ / mA cm$^{-2}$ | *FF* | *PCE* / % |
|---|---|---|---|---|
| **1** | 8.88 | 19.30 | 0.62 | 10.53 |
| **2** | 833 | 22.13 | 0.62 | 11.43 |
| **3** | 895 | 17.78 | 0.63 | 10.03 |
| **4** | 940 | 18.56 | 0.63 | 11.00 |
| **5** | 1050 | 18.07 | 0.53 | 10.06 |
| **6** | 852 | 18.30 | 0.63 | 9.82 |
| **7** | 810 | 20.66 | 0.59 | 9.87 |
| **8** | 907 | 19.49 | 0.56 | 9.90 |

**Table S3.** The photovoltaic parameters of hysteresis test for p-i-n type PSCs with various HTMs.

| HTMs | | $V_{OC}$ / mV | $J_{SC}$ / mA cm$^{-2}$ | $FF$ | $PCE$ / % |
|---|---|---|---|---|---|
| **JW6** | Forward | 850 | 19.73 | 0.61 | 10.23 |
| | Reverse | 1010 | 19.20 | 0.53 | 10.24 |
| **JW7** | Forward | 833 | 22.13 | 0.62 | 11.43 |
| | Reverse | 960 | 20.12 | 0.53 | 10.21 |
| **JW8** | Forward | 820 | 20.17 | 0.63 | 10.42 |
| | Reverse | 944 | 19.33 | 0.50 | 9.06 |
| **PEDOT** | Forward | 867 | 22.26 | 0.69 | 12.86 |
| | Reverse | 860 | 22.30 | 0.69 | 13.27 |

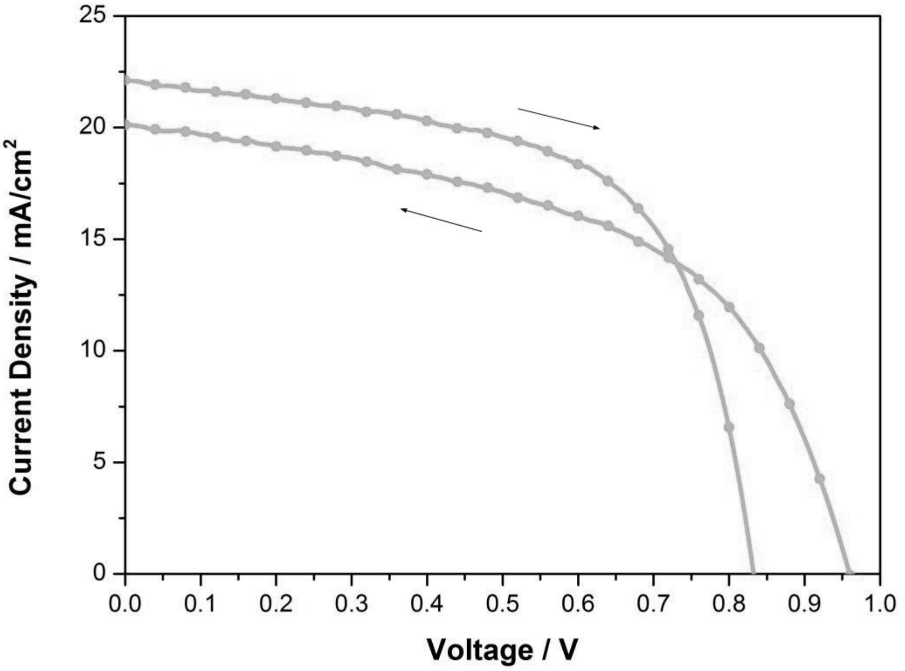


**Fig. S2.** J-V plots of p-i-n type PSCs based on **JW7** measured through reverse and forward bias at a scan rate of 0.1V/s.

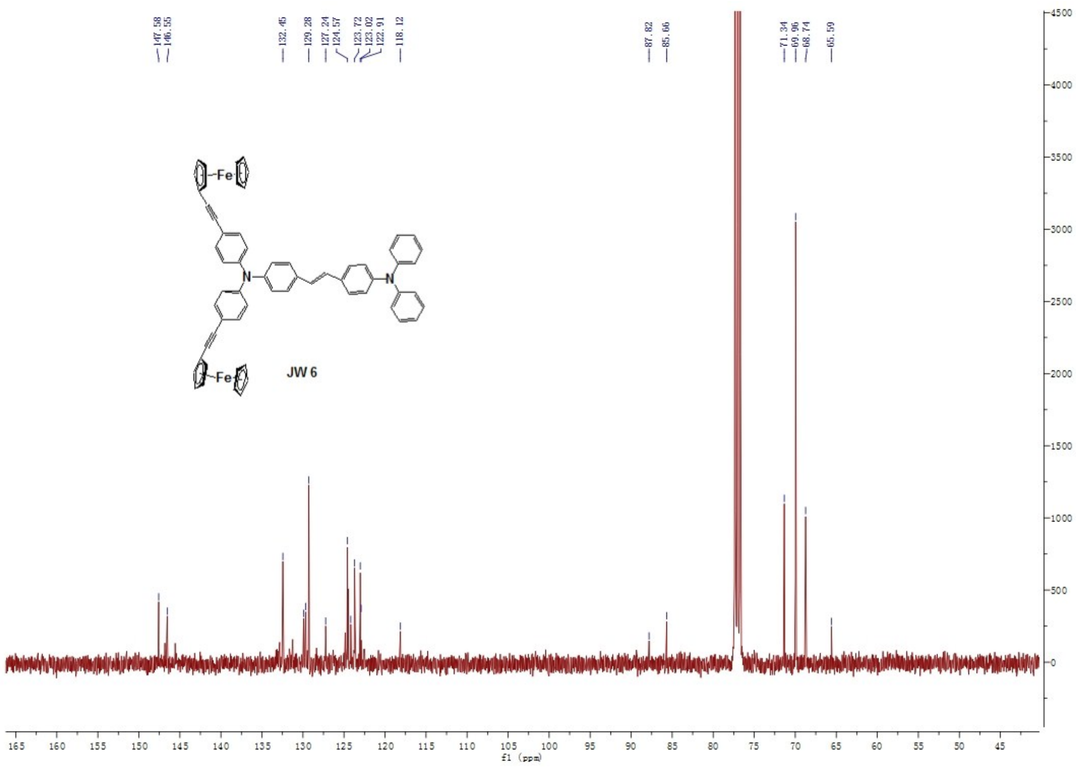

$^{1}$H NMR spectra of **JW6**

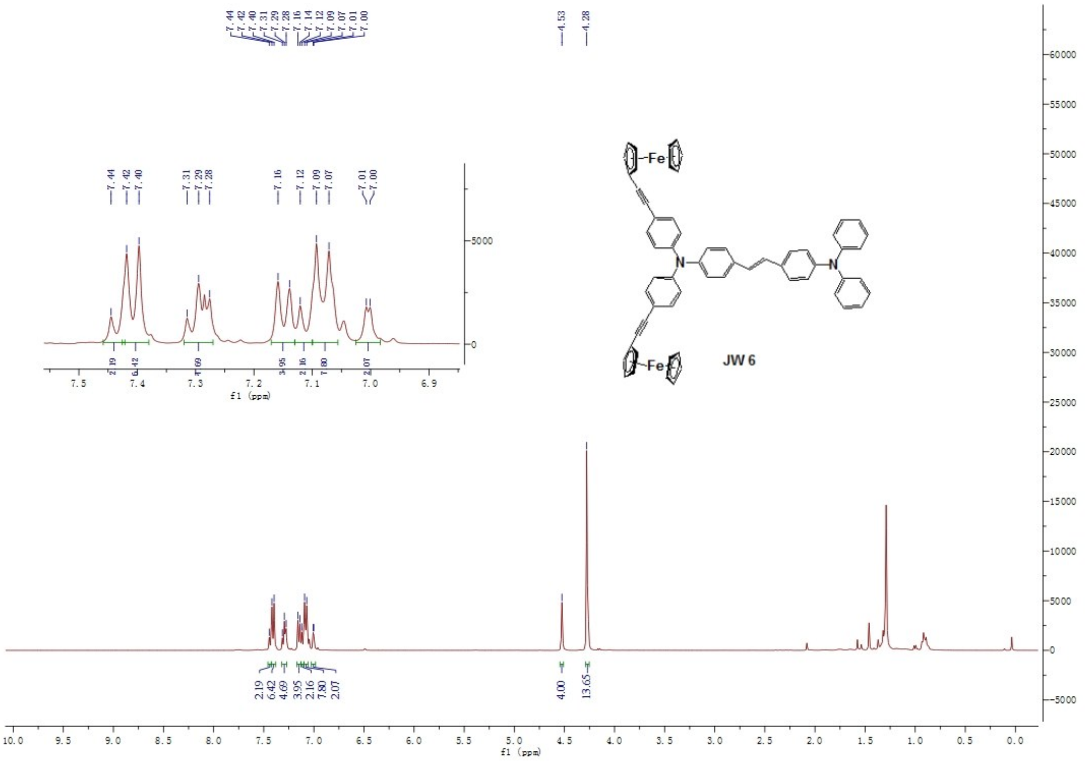

$^{13}$C NMR spectra of **JW6**

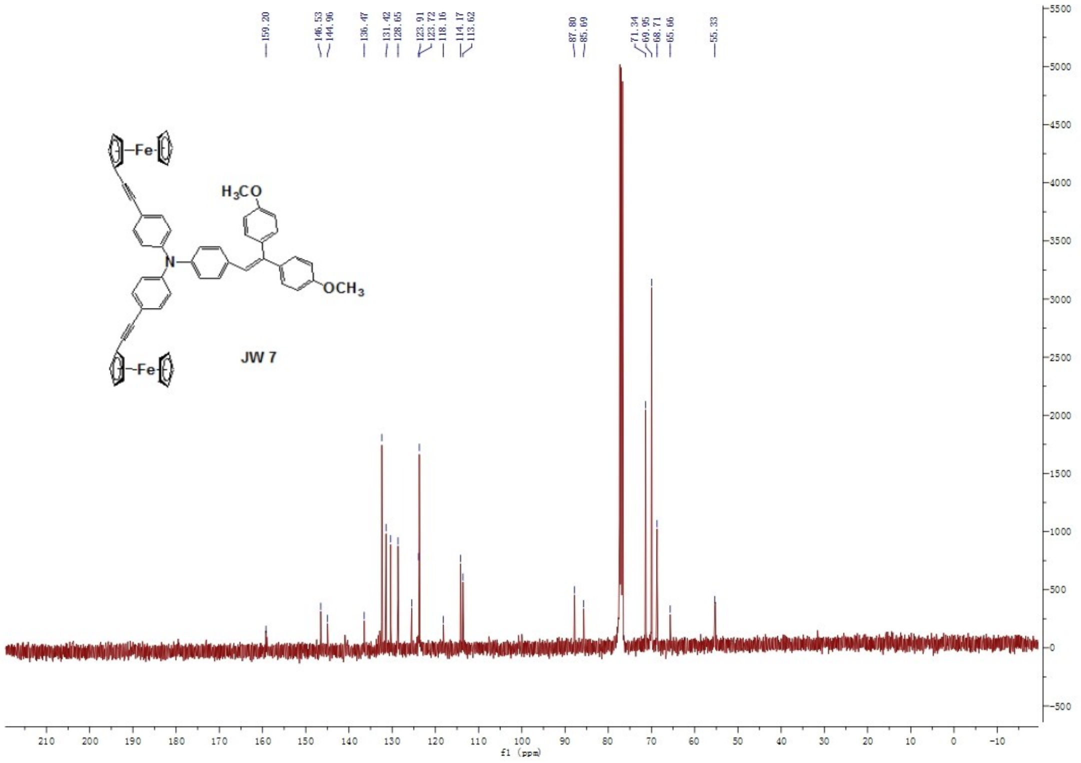


$^{1}$H NMR spectra of **JW7**

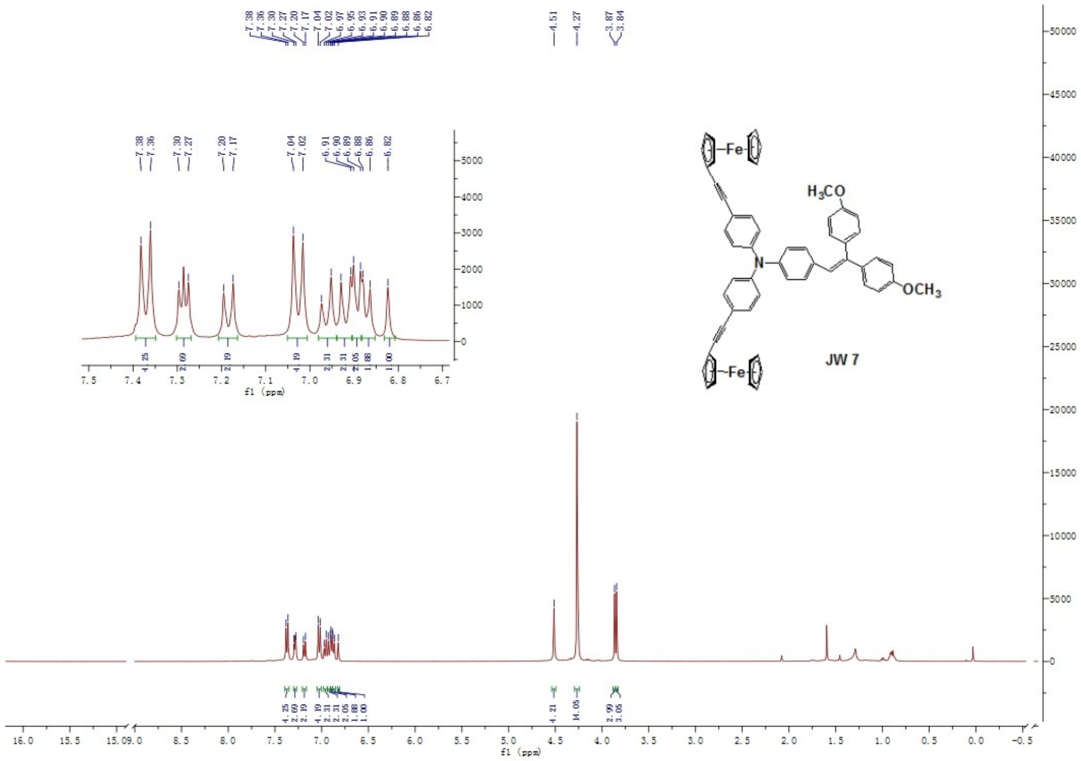


$^{13}$C NMR spectra of **JW7**

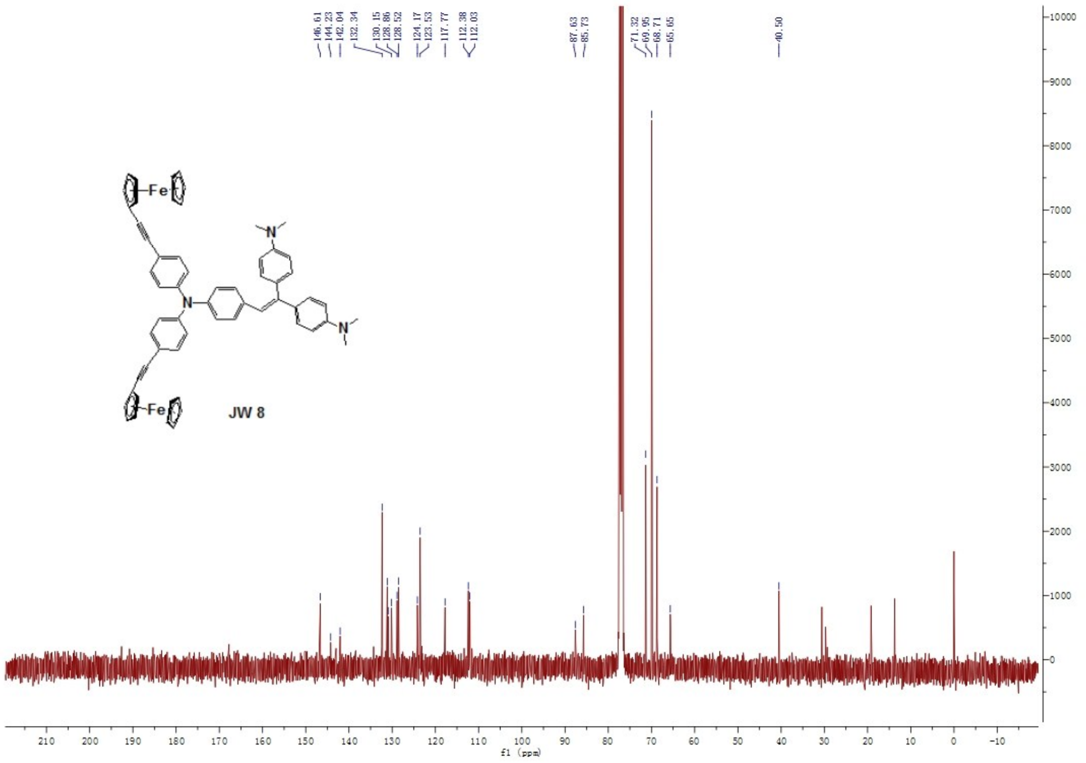


$^{1}$H NMR spectra of **JW8**

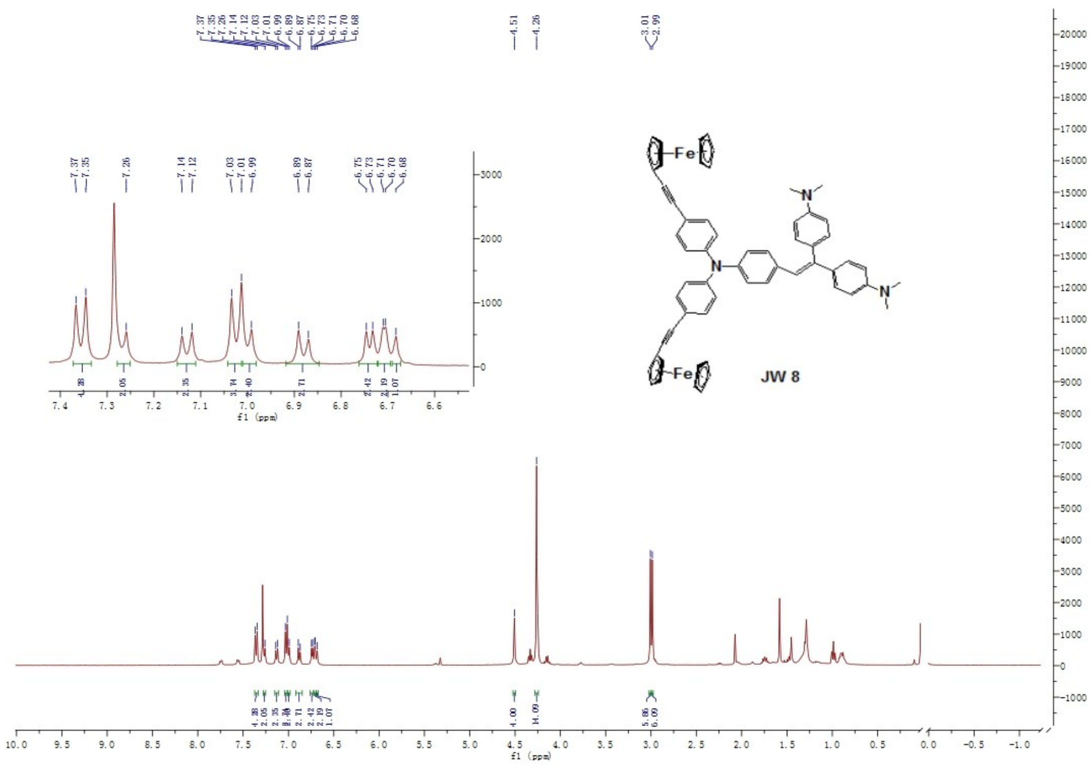


$^{13}$C NMR spectra of **JW8**
